# La proactivité dans l'adversité : L'action de la mutualité en appui des gouvernements dans la lutte contre la Covid-19

**DOI:** 10.48327/mtsimagazine.n1.2021.96

**Published:** 2021-04-28

**Authors:** T. Kanga-Tona

**Affiliations:** Chargé de Projet à l'Association Internationale de la Mutualité

**Keywords:** Mutuelles, Couverture santé universelle, Assurance santé, Protection sociale, Afrique, Covid-19, Mutual Insurance, Universal Health Coverage, Health Insurance, Social Protection, Africa, Covid-19

## Abstract

L'impact de la Covid-19 sur le continent africain a été et reste l'une des grandes inquiétudes quant aux conséquences de la pandémie. Pourtant, loin de rester passives, les mutuelles de santé ont mis en place dès le printemps 2020 une série d'actions de sensibilisation, de soutien aux populations ainsi que de soutien à l'action de leurs gouvernements. Ces actions ont pu capitaliser sur les atouts des mutuelles: un déploiement local auprès des communautés et des catégories socioprofessionnelles, mais aussi auprès des mutualistes et des personnels de centres de soins. L'action des mutuelles auprès des populations a permis de faire l'inventaire de leurs qualités, qui seront importantes dans le cadre d'une sortie équilibrée de la crise socioéconomique à venir, le vrai défi qui attend l'Afrique..

## Abstract (en)

The impact of Covid-19 on the African continent has been and remains one of the major concerns regarding the consequences of the pandemic. However, far from remaining passive, mutual health insurance companies have been implementing a series of actions since the spring of 2020 to raise awareness, support the population and support their governments’ actions. These actions capitalized on the strengths of mutual insurances: local deployment among communities and socio-professional categories, but also among mutualists and health center staff. The action of the mutual insurances with the populations has made it possible to take stock of their qualities, which will be important in the context of a balanced exit from the coming socio-economic crisis, the real challenge facing Africa.

En mai 2020, le bureau africain de l'Organisation Mondiale de la Santé (OMS) prédisait jusqu’à 190 000 victimes de la Covid-19 durant la première année de la pandémie^1^. Nous déplorons, au 25 février 2021, 101 000 morts de la Covid en Afrique^2^ ce qui est moindre que ces estimations, mais s'en rapproche. Si le continent africain compte aujourd'hui moins de malades et de morts du coronavirus que la plupart des autres régions du monde, cela ne doit pas nous faire oublier que le bilan risque de s'alourdir en fonction de l’évolution future de la maladie et ce, dans un contexte de systèmes de santé encore peu dotés en ressources.

Selon les statistiques de l'Organisation Mondiale de la Santé (OMS) et la Banque Mondiale, l'Afrique compte en moyenne 10 lits d'hôpitaux pour 10 000 personnes, comparée à 6,5 pour 1 000 personnes en France^3^, ^4^. Selon la Banque Mondiale, l'Afrique subsaharienne comptait, en 2017, 0,2 docteur pour 1 000 personnes. Dans l'Union européenne, cette moyenne était de ^3^, ^7^, ^5^. C'est dans ce contexte que la pandémie de la Covid-19 a atteint l'Afrique et a suscité les plus grandes craintes quant à son impact sur le continent. Si ces craintes ne se sont pas totalement réalisées grâce entre autres à la jeunesse de la population du continent, la précocité des mesures de confinement et de fermeture des frontières, la réactivité de la société civile ainsi qu’à des causes liées au système immunitaire de la population dans la région, la crise est le rappel qu'il est plus important que jamais de mettre sur place des systèmes de santé et de protection sociaux forts, équipés en ressources matérielles et humaines, afin de protéger au mieux les populations.

C'est dans ce contexte de systèmes de santé en constitution que les mutuelles de santé et les programmes qui les soutiennent ont mis en place des interventions en Afrique pour combattre la Covid-19. Les mutuelles promeuvent et facilitent l'accès aux services de santé à travers les mécanismes de solidarité, de prévention et de promotion de la santé, ou d'offre de services qui agissent sur déterminants sociaux de la santé. Les mutuelles sont organisées au niveau professionnel (mutuelles des agents des différentes fonctions publiques par exemple), mais sont également présentes au niveau communautaire, ce qui les place au plus près de populations qui peuvent être difficiles à atteindre par les dispositifs gouvernementaux. En intervenant en support de l'action étatique, elles ont été des alliées de fait des gouvernements dans la lutte contre la Covid-19.

L'AIM a réalisé un sondage auprès de ses membres et partenaires au cours du printemps 2020 qui a montré que les mutuelles ou les programmes qui les soutiennent ont été engagés dans des actions de lutte contre la Covid à plusieurs niveaux^6^.

Leur action s'est concentrée en majeure partie sur l'aspect préventif de la lutte contre la Covid-19, en accord avec les recommandations des autorités nationales. Ces actions ont concerné la mise en place de points de lavage des mains, la distribution de masques par exemple, appuyées par la communication auprès des mutualistes des recommandations en termes d'hygiène, des campagnes de prévention et de sensibilisation, mais également des initiatives afin de maintenir une offre minimum de soins malgré la pandémie et les mesures de restriction. Les actions ont couvert les pays suivants: Bénin, Burkina Faso, Burundi, Côte d'Ivoire, Guinée Conakry, Liban, Mali, Maroc, République Démocratique du Congo, Sénégal et Togo.

Ces actions, lancées dès mars 2020 l'ont été à destination de plusieurs types de publics: les mutualistes qui cotisent auprès des mutuelles eux-mêmes, mais également les agents des mutuelles et la population générale. Dans de nombreux cas les mutuelles ont également participé aux différents fonds de solidarité mis en place par les gouvernements ou ont offert de prendre en charge une partie des coûts de traitement de la maladie.

La crise de la Covid-19 rappelle qu'il est plus important que jamais de mettre sur place des systèmes de santé et de protection sociaux forts, équipés en ressources matérielles et humaines, afin de protéger au mieux les populations. Dans le cadre de la formulation de ses demandes post-crise Covid-19^7^, le mouvement mutualiste a précisé ses attentes, mettant l'accent sur l'importance d'adopter des piliers portant spécifiquement sur la santé et la protection sociale dans toutes les stratégies internationales de partenariats au développement en Afrique et au Moyen-Orient, afin d'atteindre les Objectifs de Développement Durable (ODD), d'investir dans les infrastructures de santé, dans la formation des professionnels de santé, dans les équipements de santé, un panier de soins, dans les systèmes d'information y compris de technologies numériques ainsi que dans les mécanismes de paiement adaptés, de prioritiser la création de systèmes d'information fiables et robustes, à commencer par les capacités de tests et de diagnostics, d'impliquer l'Afrique dans la collaboration internationale scientifique. De manière plus générale, la position de l'Afrique dans les chaînes de valeurs de produits pharmaceutiques plaide en faveur du développement d'un secteur pharmaceutique africain, particulièrement porté sur la production de médicaments génériques.

La réponse à la crise socio-économique qui menace l'Afrique reste le plus grand défi qui attend le continent. Certaines des mesures préventives prises par les gouvernements dans le cadre de la lutte contre le coronavirus ont mené à la fermeture immédiate, ou la baisse de fréquentation des centres d'accueil mutualistes. Cela a eu un impact sur la capacité de traitement des dossiers des mutualistes, qu'ils soient de remboursement, de prise en charge ou bien de nouvelles inscriptions. Si certaines mutuelles ont pu se tourner vers des solutions numériques, le développement de celles-ci au service de la dynamique mutualiste est à présent une priorité. Ces solutions permettront d'assurer la modernisation et la sécurisation des activités de collecte et de versement des cotisations, de facturation et de paiement des prestataires de soins, etc… Il faudra entreprendre un travail avec les partenaires et les États afin que tous aient accès à ces solutions techniques qui soutiennent la solidarité, et que leur accès soit assuré partout (même en zones rurales).

Les mesures nationales prises afin de réduire la propagation du virus ont également créé un tarissement des revenus des ménages. La baisse des flux de commerce international a également réduit les revenus des entreprises exportatrices, les poussant à répercuter cette baisse sur les salaires. Étant donné que la capacité de cotisation dépend de la perception d'un revenu stable et suffisant par les ménages, ce phénomène menace ainsi clairement la viabilité des mutuelles rurales qui dépendent des contributions. L'offre de soins, ainsi que la possibilité des mutuelles à se battre contre la Covid-19 sont donc à risque. Notons également que les difficultés rencontrées par les mutuelles affectent la confiance des populations dans les mécanismes d'assurance santé, y compris étatiques, à une période où, justement, un certain nombre de pays souhaitent les développer.

La réalisation de la couverture médicale universelle est et reste un défi majeur pour les sociétés du monde entier et le coronavirus prouve ce que les mutualistes disent depuis longtemps: Le manque d'investissement dans les systèmes de santé coûte cher et il est important de structurer des systèmes de santé performants et durables. Si la riposte contre la pandémie a été déterminante, la relance le sera tout autant et il faudra soutenir et développer les systèmes de soins et socioéconomiques durables après la crise. Dans de nombreux pays africains, seuls 10 % de la population ont accès aux soins de santé. Pour les travailleurs de l’économie informelle et des zones rurales, la seule possibilité est de s'assurer par le biais de mutuelles. En parallèle de la consolidation des revenus fiscaux des États africains, le volume des flux d'aide internationale doit être maintenu afin de soutenir les programmes de développement social. Alors que l'importance du droit à l'accès à des soins de santé abordables et de qualité pour toutes et tous est reconnue, passer de la théorie à la pratique n'est pas une tâche aisée. Les mutuelles relèvent ce défi majeur et préconisent de faire le « Le pari de la Mutualité », au cœur de la Plateforme de Lomé^8^. Un pari auquel les États et les organisations internationales souscrivent encore trop timidement.

